# Drunkard’s Epilepsy

**Published:** 1886-11

**Authors:** 


### Drunkard's Epilepsy.

The *Medical News* (July 31) tells us that in view of Magnin's assertion that in France the frequent cases of epilepsy occurring in drunkards are due, not to alcohol but to absinthe, Moeli has reviewed the German statistics of the subject, which may be thus summarized:

In Germany, thirty-six to forty per. cent. of the subjects of delirium tremens are also victims of epileptic attacks. An attempt to determine whether the occurrence of such attacks was correlated with the abuse of any special kind of distilled liquor was unsuccessful, but it was found that in twenty-six almost exclusively beer and wine drunkards only one was epileptic.

A CIGAR contains acetic, carbolic, formic, butyric, valeric, prussic, and propionic acids, also creasote, ammonia, sulphuretted hydrogen, pyridine, veridine, picoline, and rubidene, to say nothing of cabbagine and burdockic acid. That's why you can't get a good one for less than five cents.

